# As effective as the teacher, but less motivating: a randomized controlled comparison of AI-generated and teacher feedback on students’ writing performance and intrinsic motivation

**DOI:** 10.3389/fpsyg.2026.1918748

**Published:** 2026-10-30

**Authors:** Ying Liu, Mo Shi

**Affiliations:** 1 School of Education, Fuzhou University of International Studies and Trade, Fuzhou, China; 2 School of Economics and Management, Ankang University, Ankang, China

**Keywords:** artificial intelligence, automated feedback, intrinsic motivation, randomized controlled experiment, self-determination theory, writing instruction

## Abstract

**Introduction:**

Generative artificial intelligence (AI) offers scalable feedback on student writing; however, its effects on writing performance and motivation relative to teacher feedback remain insufficiently understood. Drawing on Feedback Intervention Theory and Self-Determination Theory, this study examined the comparative effectiveness of AI-generated and teacher-provided feedback on students’ writing performance and motivational outcomes.

**Methods:**

A randomized controlled study assigned 312 undergraduate students to one of three conditions: AI-generated feedback, teacher feedback, or a control condition without substantive feedback. A total of 287 participants were included in the analyses. The study evaluated revised-essay quality, feedback engagement, writing self-efficacy, intrinsic motivation, perceived relatedness to the feedback source, and AI literacy.

**Results:**

Both AI-generated feedback and teacher feedback produced higher revised-essay quality than the control condition, with effect sizes of *d* =  0.60 and *d* = 0.59, respectively. The two feedback conditions were statistically equivalent within a prespecified equivalence margin of ±0.35 standard deviations. Feedback engagement, but not writing self-efficacy, statistically accounted for the observed performance advantage. Teacher feedback elicited higher intrinsic motivation than AI-generated feedback, although AI-generated feedback also yielded higher intrinsic motivation than the control condition. This motivational difference was associated with perceived relatedness to the feedback source. The indirect effects identified in the analyses are associational rather than causal. AI literacy did not significantly moderate the effect of feedback on writing performance (*p* = 0.058).

**Discussion:**

Within the context of a single feedback-and-revision cycle at one institution, the findings suggest that AI-generated feedback can achieve writing-performance outcomes comparable to those of teacher feedback, while providing weaker motivational support. These results highlight the potential value of blended feedback approaches that combine the scalability of AI-generated feedback with the relational and motivational strengths of teacher–student interaction. Further research across multiple institutions and repeated feedback cycles is needed to establish the generalizability and longer-term implications of these findings.

## 1 Introduction

Feedback is among the most consequential influences on student learning, but its power has always been constrained by a stubborn practical limit: skilled human feedback is slow, effortful, and scarce. The arrival of generative artificial intelligence (AI) appears to dissolve that constraint. Large language models can now read a student essay and return paragraphs of specific, fluent, criterion-referenced commentary in seconds, at negligible marginal cost (Kasneci et al., 2023). Within 2 years of these tools becoming publicly available, students and instructors began using them to generate writing feedback at scale (Chan and Hu, 2023; Cotton et al., 2023; Dwivedi et al., 2023; Tlili et al., 2023). The speed of adoption has far outpaced the evidence base, leaving educators with a seemingly simple question: when a machine gives a student feedback on their writing, is it as good as feedback from their teacher?

The question is deceptively simple because “as good as” conceals at least two distinct criteria. The first is instrumental: does the feedback help students write better? The second is motivational: does the feedback sustain students’ desire to keep writing and learning? These criteria need not move together. A long tradition in educational psychology warns that feedback can raise performance while depressing motivation, or vice versa, depending on how learners interpret its source and intent (Kluger and DeNisi, 1996; Ryan and Deci, 2000). Feedback from a teacher is never only information; it is also a social act that signals attention, care, and relationship. Feedback from an algorithm carries the information but, arguably, not the relationship. Whether that difference matters for learning is an open and pressing empirical question.

Existing research on AI writing feedback has grown quickly but remains lopsided. Most studies evaluate the quality or accuracy of AI-generated feedback by comparing it against human raters or rubrics (Steiss et al., 2024), or document students’ perceptions and preferences (Escalante et al., 2023). Far fewer test, under experimental control, whether AI feedback actually changes what students produce relative to a no-feedback baseline, and fewer still examine motivational outcomes alongside performance. The field has also paid limited attention to mechanism: even where AI feedback “works,” we rarely know why, or for whom. These gaps matter because policy decisions are already being made, schools and universities are deciding whether to fold AI feedback into writing instruction, and they are doing so largely in the dark about its effects on the affective side of learning, even as institutions race to issue policy guidance on its use (Chan, 2023; Chiu, 2023b).

The present study addresses these gaps with a three-arm randomized controlled experiment that compares AI-generated feedback, teacher feedback, and a no-substantive-feedback control on undergraduates’ argumentative writing. We make three contributions. Theoretically, we integrate Feedback Intervention Theory (Kluger and DeNisi, 1996), which explains how feedback shapes performance through the cognitive processing it elicits, with Self-Determination Theory (Ryan and Deci, 2000), which explains how the social meaning of feedback shapes motivation. Considering these two channels jointly yields a dual-pathway prediction: that AI and teacher feedback should converge on performance, because the cognitive channel is indifferent to source, yet diverge on motivation, because the relational channel is not. Empirically, we use an equivalence-testing framework to ask not merely whether AI and teacher feedback differ, but whether AI feedback is good enough to be considered practically equivalent to a teacher on performance, a question conventional null-hypothesis tests cannot answer. Methodologically, we examine both mediating mechanisms (engagement, self-efficacy, relatedness) and a theoretically motivated boundary condition (students’ AI literacy), moving beyond “does it work” to “how and for whom.” The remainder of the paper develops the theoretical framework and hypotheses, reports the experiment, and discusses what a performance-motivation dissociation implies for the design of feedback in an age of capable machines.

## 2 Theoretical background and hypotheses

### 2.1 Feedback, processing, and writing performance

Feedback is information about the gap between a current and a desired level of performance, but information alone does not improve learning. In their foundational meta-analysis and the Feedback Intervention Theory (FIT) they derived from it, Kluger and DeNisi (1996) showed that feedback interventions vary enormously in their effects, and that a sizable minority actually harm performance. The decisive factor is where feedback directs the learner’s attention. Feedback that focuses attention on the task and on the cognitive operations needed to improve it tends to help; feedback that diverts attention to the self tends to hurt. This insight reframes feedback effectiveness as a question of processing: feedback works to the extent that it provokes the learner to engage with, interpret, and act on it (Alt et al., 2023; Black and Wiliam, 2009; Carless and Boud, 2018). Contemporary accounts of feedback as a learner-centered process make the same point, arguing that the active ingredient is the student’s engagement with comments rather than the comments themselves (Bond et al., 2020; Molloy et al., 2019; Nicol, 2020; Sadler, 2010).

In writing specifically, this processing view is well supported. Students who engage more deeply with feedback, attending to it, evaluating it, and using it to guide revision, produce stronger revised texts (Ferris and Roberts, 2001; Zhang and Hyland, 2018). Importantly, the source of feedback may matter less for this cognitive pathway than its substance: if AI-generated comments are specific, relevant, and actionable, they should be capable of provoking the same depth of engagement as a teacher’s comments, and therefore the same revision gains. This reasoning leads to our first, deliberately inclusive hypothesis: that substantive feedback of either kind outperforms no feedback. Functionally, H1 also serves as a positive control, establishing that the feedback streams are potent enough to make the subsequent equivalence test meaningful.

*H1:* Relative to a no-feedback control, both AI-generated feedback and teacher feedback produce higher writing performance on a revised text.

### 2.2 Is AI feedback as good as a teacher? An equivalence question

The more provocative question is comparative. Early evidence on AI writing feedback is genuinely mixed. Some studies find that large language models generate feedback rated as comparable to, or in places more detailed than, that of trained human raters (Mahapatra, 2024; Steiss et al., 2024), and that students often perceive AI feedback as useful and prefer its immediacy (Baskara, 2023; Escalante et al., 2023; Xiao and Zhi, 2023). Others document shortcomings, AI feedback can be generic, occasionally inaccurate, and weaker on higher-order rhetorical concerns than human feedback (Banihashem et al., 2024; Marzuki et al., 2023). At the same time, early intervention studies suggest that AI writing tools can support not only text quality but also writing self-efficacy and motivation (Nazari et al., 2021; Song and Song, 2023; Tran, 2025; Yılmaz and Yılmaz, 2023), even as broad reviews caution that the pedagogical and ethical implications of large language models in education remain only partly mapped (Chiu, 2023a; Yan et al., 2023). Emerging evidence further warns that uncritical reliance on generative AI can encourage shallow, metacognitively lazy processing (Fan et al., 2024), underscoring that how student-AI interaction is designed shapes whether it helps or harms learning (Kim et al., 2022). Quasi-experimental and correlational designs dominate this literature, and most studies benchmark AI feedback against humans on feedback quality rather than on the learning that the feedback produces (Crompton and Burke, 2023; Zawacki-Richter et al., 2019).

Critically, the practical question facing educators is not whether AI feedback differs from teacher feedback by some non-zero amount, which, given sufficient power, it almost certainly does, but whether any difference is small enough to be educationally negligible. This is a question of equivalence, not of difference, and it cannot be answered by a non-significant conventional test, which conflates “no evidence of a difference” with “evidence of no difference” (Lakens et al., 2018). We therefore frame the AI-versus-teacher comparison on performance as an equivalence hypothesis, predicting that AI feedback falls within a margin of practical equivalence to teacher feedback. This equivalence is conditional rather than absolute: Feedback Intervention Theory implies that when AI and teacher comments are matched on specificity and actionability, as our design enforces, they should provoke comparable processing and therefore comparable performance. The theory thus explains why matched content yields matched performance rather than predicting that source is irrelevant in general.

*H2:* AI-generated feedback is statistically equivalent to teacher feedback in its effect on writing performance.

### 2.3 Mechanisms of the performance effect: engagement and self-efficacy

If feedback improves writing, two mechanisms are commonly proposed. The first, following FIT and feedback-as-process accounts, is engagement: feedback prompts the learner to process the gap between current and desired performance and to undertake the cognitive work of revision (Carless and Boud, 2018; Sweller et al., 2019; Zhang and Hyland, 2018). The second is motivational-cognitive: feedback may raise a learner’s writing self-efficacy, the belief that one can execute the actions needed to write well, which in turn supports performance (Bandura, 1977; Schunk, 1991). These mechanisms make different predictions about source. Engagement should respond to the quantity and specificity of feedback, attributes on which AI excels, whereas self-efficacy, which is nourished partly by credible social persuasion from a valued other, may respond more to who delivers the feedback. We test both as parallel mediators of the performance effect, expecting engagement to carry the effect robustly and remaining agnostic, but attentive, to the role of self-efficacy.

*H3:* The effect of feedback on writing performance is mediated by (a) depth of feedback engagement and (b) writing self-efficacy.

### 2.4 The social meaning of feedback: source, relatedness, and motivation

Performance is only half the story. Self-Determination Theory (SDT) holds that the quality of a learner’s motivation depends on the satisfaction of three basic psychological needs, autonomy, competence, and relatedness, and that the interpersonal context of an event shapes which needs it satisfies or thwarts (Niemiec and Ryan, 2009; Ryan and Deci, 2000; Vansteenkiste et al., 2020). Feedback is precisely such an event. When feedback comes from a teacher, it is embedded in a relationship: it signals that someone knowledgeable has attended to the student’s work and cares about its improvement, supporting relatedness and, through it, intrinsic motivation. When the identical informational content comes from an algorithm, the relational signal is attenuated. The student may find the comments useful, but is unlikely to experience them as an act of care from a valued other.

This source asymmetry has begun to surface in studies of human-machine interaction, where people extend less trust and ascribe less benevolence to automated agents than to humans even when the agents perform competently (Adam et al., 2020; Araujo et al., 2020; Blut et al., 2021). In education, emerging work hints that students value the efficiency of AI feedback while still craving the human connection of teacher feedback (Barrett and Pack, 2023; Chan and Hu, 2023). Synthesizing SDT with this evidence, we predict that teacher feedback fosters higher intrinsic motivation than AI feedback, and that perceived relatedness to the feedback source appears to carry this difference. Notably, this is a prediction about motivation that runs orthogonal to the equivalence we expect on performance, the theoretical crux of our integration.

*H4:* Teacher feedback produces higher intrinsic motivation than AI-generated feedback, and this difference is mediated by perceived relatedness to the feedback source.

### 2.5 A boundary condition: AI literacy

Finally, the benefit a student derives from AI feedback is unlikely to be uniform. Making productive use of machine-generated comments, judging their credibility, interpreting their intent, and deciding which to act on, is itself a competence. AI literacy, the knowledge and skills needed to understand, evaluate, and interact with AI systems, has been conceptualized and measured as exactly such a competence (Carolus et al., 2023; Ng et al., 2023; Wang et al., 2022). Students higher in AI literacy should be better positioned to extract value from AI feedback, engaging with it more critically and translating it into revision more effectively, whereas the value of teacher feedback, a familiar genre, should depend little on AI literacy. We therefore predict that AI literacy moderates the effect of AI feedback specifically.

*H5:* AI literacy moderates the effect of AI feedback on writing performance, such that the benefit of AI feedback is larger for students higher in AI literacy.

Figure 1 summarizes the hypothesized model, in which feedback condition influences performance through engagement and self-efficacy and influences motivation through relatedness, with AI literacy moderating the AI-feedback pathway.

**Figure 1:**
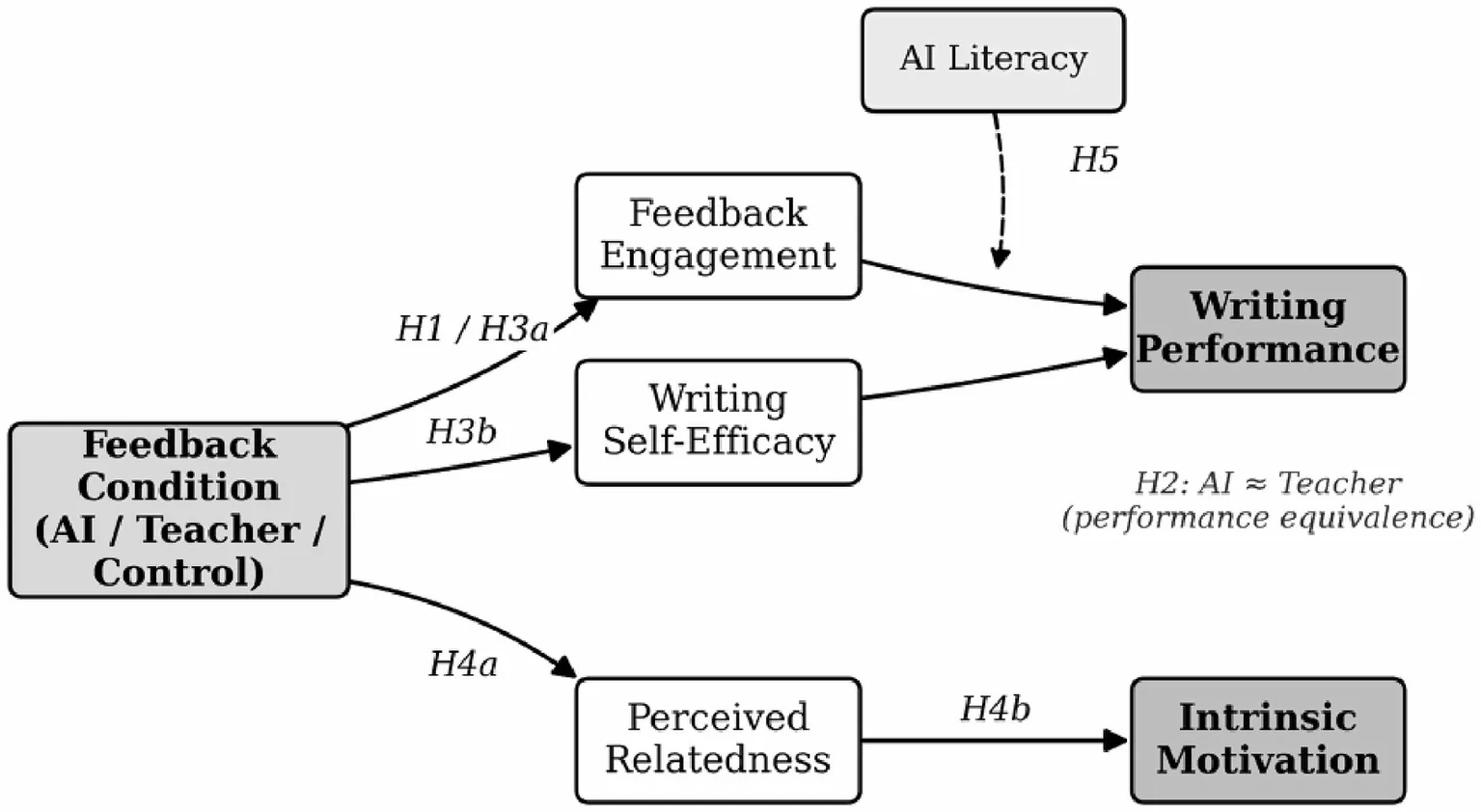
Hypothesized conceptual model. Feedback condition influences writing performance through feedback engagement and writing self-efficacy, and influences intrinsic motivation through perceived relatedness. AI literacy moderates the AI-feedback pathway to performance. H1–H5 denote the hypotheses.

## 3 Methods

### 3.1 Design and participants

We employed a three-arm, parallel-group randomized controlled experiment (Deaton and Cartwright, 2017) with feedback source as the between-subjects factor (AI-generated feedback vs. teacher feedback vs. no-substantive-feedback control) and a pre-test measure of writing as a covariate. An *a priori* power analysis using G*Power (Faul et al., 2007) indicated that detecting a medium between-condition difference (*f* = 0.20) in a three-group fixed-effects model with one covariate, at *α* = 0.05 and power = 0.80, required a minimum of 246 participants; we over-recruited to allow for attrition. This *a priori* analysis addressed the omnibus between-condition comparison, not the equivalence test; a separate power analysis for the equivalence test is reported in Section 3.4. The study was not preregistered. The equivalence margin of d = 0.35 was determined *a priori* on theoretical and practical grounds (see Section 3.4) before data collection began.

Of 340 undergraduate students assessed for eligibility at a comprehensive university, 16 did not meet the inclusion criteria (enrolled in a first-year academic-writing course, native or near-native proficiency, no prior use of AI feedback in the course) and 12 declined to participate; the remaining 312 were randomized (AI = 104, teacher = 102, control = 106). Twenty-five participants were lost to follow-up or excluded for failing an attention check or not completing the revision task (AI = 8, teacher = 8, control = 9; 3 failed the embedded attention check and 5 did not complete the revision in the AI group; 4 and 4, respectively, in the teacher group; 3 and 6 in the control group), yielding a final analytic sample of *N* = 287 (AI = 96, teacher = 94, control = 97; attrition = 8.0%, or 7.7%, 7.8%, and 8.5% by arm, *χ*^2^(2) = 0.05, *p* = 0.975); Figure 2 details the flow of participants through the study. Participants averaged 20.40 years of age (*SD* = 1.96), and 55.4% identified as women; mean grade-point average was 3.03 (*SD* = 0.58). The three conditions did not differ on pre-test writing, GPA, age, or gender distribution (all ps > 0.38; see Section 4.1), consistent with successful randomization.

**Figure 2:**
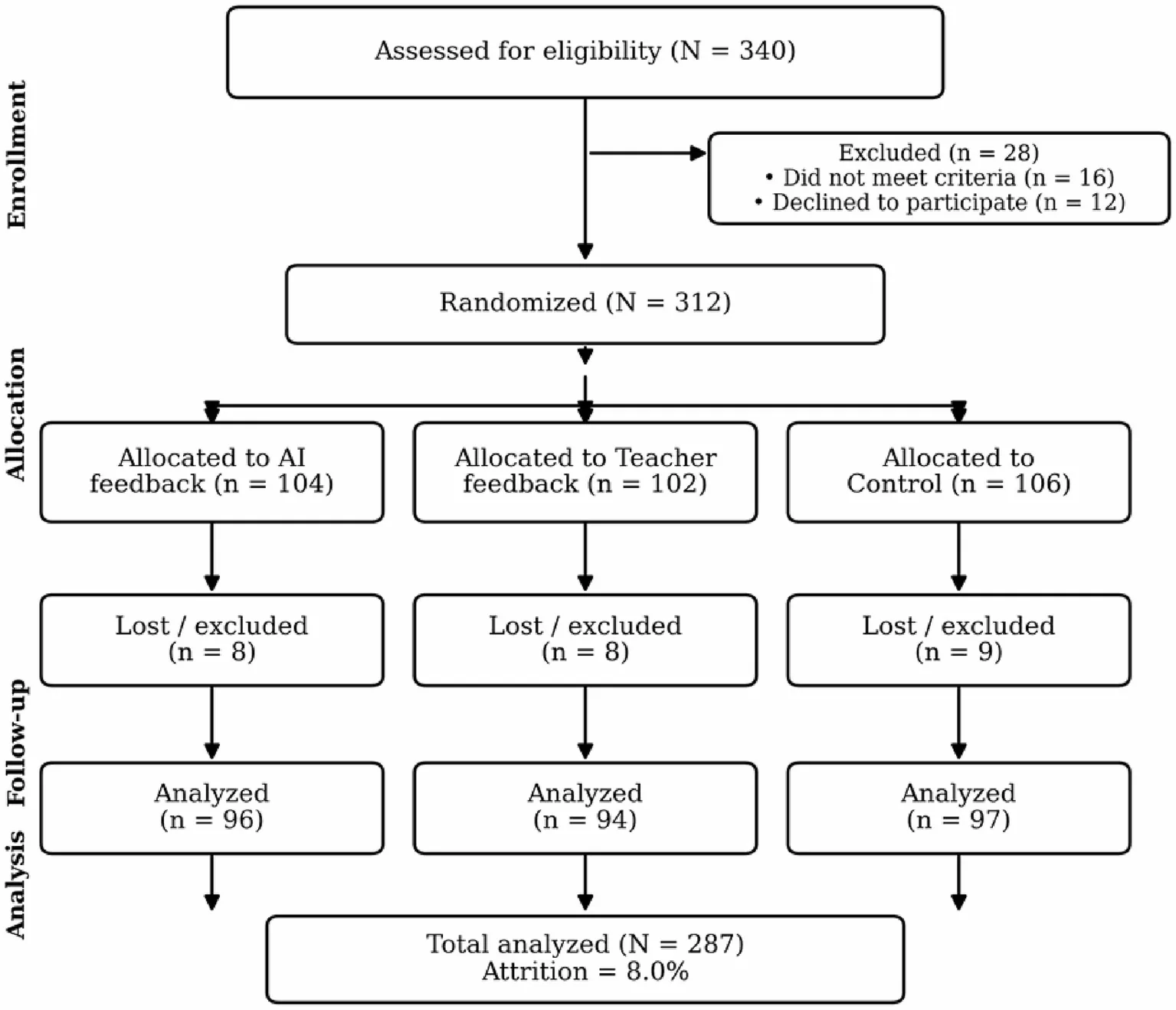
Participant flow diagram (CONSORT). Flow of participants through enrollment, allocation, follow-up, and analysis. Reasons for loss or exclusion by arm: AI feedback, 3 failed the attention check and 5 did not complete the revision; teacher feedback, 4 and 4; control, 3 and 6. All analyses were complete-case analyses of the participants shown as analyzed.

*Randomization and allocation concealment.* Participants were individually randomized by lottery, using simple randomization with an equal (1:1:1) allocation probability. Before data collection, a research assistant with no other role in the study generated the allocation sequence with a computer random-number generator, wrote each condition code (A = AI feedback, B = teacher feedback, C = control) on an identical card, and sealed the cards in opaque envelopes. The experimenters enrolled participants at the start of Session 1: after giving consent, each participant drew one envelope from a box and handed it in, unopened, with the pre-test essay, so that neither the participant nor the experimenters knew the assignment until the first draft was complete. Because randomization was simple rather than blocked, the arms were of slightly unequal size. The two raters who scored the essays did not know the condition of any essay (Section 3.3). Students could not be blinded to their condition, because the source of their feedback was disclosed to them (see Section 5.5).

*Ethics and consent.* All procedures were approved by the Ethics Review Committee of Guangzhou Municipal Institute of Educational Sciences (Approval No. GIES-ERC-3245; approved 20 August 2025). Participation was voluntary, and students provided informed consent before any data were collected. Consent was given in writing, and students were told that they could withdraw at any time without penalty to their course standing. Students who took part received research-participation credit equivalent to 2% of the course grade for attending both sessions; the credit did not depend on essay performance or on the condition to which a student was assigned. An equivalent reflective-writing exercise carrying the same credit was available to students who chose not to participate.

### 3.2 Procedure and feedback manipulation

The experiment was conducted across two class sessions one week apart. In Session 1 (approximately 60 min), participants provided informed consent, completed a demographic questionnaire and the AI-literacy scale, and wrote their pre-test argumentative essay on a common prompt (see below). Over the following five days, the instructor wrote individualized feedback for participants assigned to the teacher-feedback condition; AI feedback for the AI-feedback group was generated on the day of Session 1 using the locally deployed language model (see below).

The essay prompt was: “Should universities require all students to take a course in artificial intelligence? Write an argumentative essay of approximately 400 words presenting and defending your position.” Participants had 45 min to write approximately 400 words; this constituted the pre-test draft.

In Session 2, held one week after Session 1 and lasting approximately 70 min, each participant received their assigned feedback, printed together with a copy of their own draft, or, in the control condition, the content-neutral prompt described below, and had 15 min to read it. Participants then completed the process and perception measures and revised their essay, as described at the end of this section.

In the AI-feedback condition, the pre-test draft was submitted to a large language model operating from a standardized rubric-based prompt, which returned written feedback addressing thesis, argument structure, evidence, and clarity. The model was Qwen2.5-27B-Instruct (Alibaba Cloud), deployed locally on the research team’s server and accessed via its REST API. All feedback was generated in September 2025 with the temperature set to 0.7 and maximum output length set to 1,024 tokens. The complete prompt is reproduced in Appendix B. Student names and all identifying information were removed from each essay before it was submitted to the model. Because the model was deployed on the research team’s own server, no essay data were transmitted to any external provider and no data-retention or model-training concerns applied. Each essay was submitted in a fresh context with no conversation history, so that feedback on one essay could not influence feedback on another. The AI-generated feedback was reviewed by two members of the research team for appropriateness; no output was edited or regenerated.

In the teacher-feedback condition, the feedback was written by one instructor who held a Master’s degree in Applied Linguistics and had six years of experience teaching English academic writing at the university level. The instructor was trained on the same rubric (a two-hour calibration session in which the instructor scored and discussed five sample essays with the research team using the study’s analytic rubric) and produced written feedback of comparable length on the same criteria, spending approximately 15 min per essay. The instructor was blind to the study hypotheses, to students’ pre-test scores, and to the existence of the AI-feedback condition.

In the control condition, participants received only a generic completion message and a content-neutral prompt to “review your essay,” with no substantive commentary. Control participants were given the same fixed reading period as the two feedback conditions (15 min), during which they re-read their own draft, so that all three groups spent the same amount of time with their essay before revising. The control therefore held time on task constant, but it differed from the feedback conditions in two respects that this design cannot separate: students in the feedback conditions received comments on their own essay, and, because those comments were organized around the rubric criteria, they were also reminded of what the criteria were. The advantage of the feedback conditions over the control is therefore best read as the combined benefit of essay-specific, rubric-referenced commentary relative to minimal prompting; separating the two would require an additional control condition that presented the rubric criteria without essay-specific comments. Feedback length and formatting were equated across the two feedback conditions to prevent confounding feedback source with feedback volume (mean length: AI = 304 words; teacher = 291 words).

After reading their feedback, participants completed the process and perception measures (approximately 10 min) and then revised their essay (the post-test draft), with 40 min allowed for revision. The revised essays were scored later by two trained raters, as described below.

### 3.3 Measures

Unless otherwise noted, multi-item constructs were measured on seven-point Likert scales (1 = strongly disagree, 7 = strongly agree), and scale scores were computed as the mean of available items.

Writing performance. The pre- and post-test essays were scored on a 100-point analytic rubric (thesis, organization, evidence, and language; the full rubric, with band descriptors and point allocations, is reproduced in Appendix A) by two trained raters blind to condition and to whether a draft was a pre- or post-test. The raters were two experienced academic-writing instructors (both holding Master’s degrees in English Education, with four and five years of teaching experience, respectively), neither of whom was the instructor who provided the teacher feedback, and completed a four-hour calibration workshop in which they independently scored 20 sample essays, discussed discrepancies, and re-scored until inter-rater agreement on the training set exceeded 0.85 before scoring. Inter-rater reliability was good (ICC = 0.82; two-way random-effects model, absolute agreement, averaged across the two raters—ICC(2,2)—computed across all pre- and post-test essays), and the two raters’ scores were averaged.

Feedback engagement. Five items adapted from work on student engagement with feedback (Zhang and Hyland, 2018) assessed the depth with which students processed and acted on their feedback (e.g., “I thought carefully about the feedback before revising”); Cronbach’s *α* = 0.81.

Writing self-efficacy. Five items based on academic self-efficacy formulations (Bandura, 1977; Schunk, 1991) captured confidence in executing key writing tasks (e.g., “I am confident I can organize my arguments effectively”); *α* = 0.84.

Perceived relatedness. Four items adapted from Self-Determination Theory measures (Ryan and Deci, 2000) assessed the sense of connection to the feedback source (e.g., “The feedback made me feel that someone cared about my writing”); *α* = 0.82.

Perceived usefulness. Four items assessed the instrumental value attributed to the feedback (e.g., “The feedback gave me concrete ideas for improvement”); *α* = 0.81.

Intrinsic motivation. Six items adapted from the interest-enjoyment subscale of the Intrinsic Motivation Inventory (Ryan and Deci, 2000) measured intrinsic motivation toward the writing task (e.g., “I would enjoy working on a task like this again”); *α* = 0.86.

AI literacy. Six items drawn from validated AI literacy instruments (Carolus et al., 2023; Ng et al., 2023; Wang et al., 2022) assessed students’ competence in understanding and working with AI tools; *α* = 0.85. AI literacy was measured at baseline, prior to randomization.

Covariates. We recorded age, gender, GPA, and prior frequency of AI-tool use.

### 3.4 Analytic strategy

We first examined randomization success, descriptive statistics, and the measurement model. The six latent constructs were evaluated with confirmatory factor analysis (CFA); internal consistency was indexed by Cronbach’s *α*, interpreted following Taber (2017); and common method variance was assessed with Harman’s single-factor test (Podsakoff et al., 2003). Measurement invariance of the six-factor model across the three conditions was examined with multi-group CFA, comparing configural, metric (equal loadings), and scalar (equal loadings and intercepts) models; a decrease in CFI of no more than 0.010, supplemented by increases of no more than 0.015 in RMSEA and 0.030 (metric) or 0.010 (scalar) in SRMR, was taken to indicate invariance (Chen, 2007). The primary performance hypothesis (H1) was tested with analysis of covariance (ANCOVA) on post-test writing, controlling for pre-test writing, GPA, and gender, with planned contrasts of each feedback condition against control. ANCOVA assumptions were examined by testing the homogeneity of regression slopes (condition × pre-test interaction) and the homogeneity of residual variances (Brown–Forsythe version of Levene’s test), by inspecting the distribution of residuals, standardized residuals, and Cook’s distances, and by re-estimating the planned contrasts with heteroskedasticity-consistent (HC3) standard errors. To control the familywise error rate within each family of planned comparisons (the two feedback-versus-control contrasts on performance and the three pairwise contrasts on motivation), *p* values were adjusted with the Holm (1979) procedure. The equivalence of AI and teacher feedback on performance (H2) was tested with a two one-sided tests (TOST) procedure (Lakens et al., 2018). We set the smallest effect size of interest *a priori* at *d* = 0.35, before the equivalence analysis was conducted, corresponding to roughly 3.7 rubric points (with *d* standardized on the covariate-adjusted residual *SD* of 10.62 points), about one third of a 10-point letter-grade band, below which a difference between feedback sources would be of little practical consequence for instruction. Two further considerations informed this choice. First, a difference of 3.7 points is smaller than the standard error of measurement of an individual essay score in this study (12.26 × √(1–0.82) ≈ 5.2 points), so differences inside the margin are finer than the precision with which a single essay can be scored. Second, consistent with the recommendation of Lakens et al. (2018) to justify equivalence bounds by the practical consequences of an effect, we anchored the margin in the units in which instructional decisions are made (rubric points and grade bands) rather than in a generic benchmark for a small effect. Because a margin of this size cannot exclude smaller differences, we additionally examined the robustness of the equivalence conclusion to stricter margins. Because the *a priori* power analysis targeted the omnibus ANCOVA, we also computed the power of the TOST procedure for the achieved sample (*n* = 96 and 94), using the standard error of the covariate-adjusted AI-minus-teacher difference (1.55 points) and *α* = 0.05 for each one-sided test. Assuming a true difference of zero, power to establish equivalence within ±0.35 *SD* was 0.55; about 145 participants per arm would have been needed for power of 0.80, and with the achieved sample, power reached 0.80 only for a margin of about ±0.43 *SD*. Because the TOST procedure holds the probability of falsely concluding equivalence at *α* = 0.05 regardless of power, a significant result remains interpretable; limited power instead raises the risk of failing to demonstrate an equivalence that is present. Mediation hypotheses (H3, H4) were tested with percentile bootstrap analyses (5,000 resamples) of indirect effects (Preacher and Hayes, 2008): a parallel two-mediator model (engagement and self-efficacy) for the performance pathway, and a single-mediator model (relatedness) for the motivation pathway. Because the mediators were not randomized, and because relatedness and intrinsic motivation were measured on the same occasion, these models rest on untestable assumptions (in particular, no unmeasured confounding of the mediator–outcome relation); we therefore interpret the indirect effects as consistent with, rather than as demonstrating, causal mediation. The moderation hypothesis (H5) was tested with hierarchical regression, entering the AI-literacy-by-condition interaction terms after main effects and probing interactions with simple-slope analysis. Secondary models (the equivalence, mediation, and moderation tests) retained the pre-test as the sole covariate, because randomization equated the conditions on the demographic variables. Item-level missingness was low (0.7–5.9%) and was handled by computing scale scores from available items; the multi-group CFA models were estimated with full-information maximum likelihood. All analyses were complete-case analyses of the 287 participants who completed the revision task and passed the attention check; an intention-to-treat analysis of all 312 randomized participants was not conducted.

## 4 Results

### 4.1 Preliminary analyses

Randomization was successful: the three conditions did not differ on pre-test writing, *F*(2, 284) = 0.09, *p* = 0.910, GPA, *F*(2, 284) = 0.40, *p* = 0.670, age, *F*(2, 284) = 0.08, *p* = 0.919, or gender, *χ*^2^(2) = 1.90, *p* = 0.388. Table 1 reports descriptive statistics, reliabilities, and zero-order correlations among the study variables. Pre- and post-test writing were strongly correlated (*r* = 0.56), and feedback engagement related to post-test performance (*r* = 0.53), whereas relatedness related to intrinsic motivation (*r* = 0.54), patterns consistent with the hypothesized pathways.

**Table 1:** Descriptive statistics, reliabilities, and correlations among study variables.

Variable	*M*	*SD*	1	2	3	4	5	6	7	8
1. Pre-test writing	61.65	12.26	–							
2. Post-test writing	52.49	13.22	0.56	–						
3. Feedback engagement	4.41	0.85	0.08	0.53	(0.81)					
4. Writing self-efficacy	4.89	0.84	0.06	0.24	0.16	(0.84)				
5. Perceived relatedness	4.17	0.92	0.03	0.08	0.14	0.06	(0.82)			
6. Perceived usefulness	4.56	0.84	0.12	0.24	0.16	0.08	0.15	(0.81)		
7. Intrinsic motivation	4.89	0.83	0.02	0.12	0.10	0.19	0.54	0.12	(0.86)	
8. AI literacy	4.46	0.84	0.25	0.27	0.17	0.11	0.00	0.08	0.03	(0.85)

*N* = 287. Coefficient alpha is on the diagonal in parentheses. Correlations |*r*| ≥ 0.12 are significant at *p* < 0.05. Pre- and post-test writing are on a 100-point scale; all other variables use 7-point scales.

The six-factor measurement model fit the data well: *χ*^2^(390) = 550.85, *χ*^2^/*df* = 1.41, CFI = 0.952, TLI = 0.946, RMSEA = 0.038, SRMR = 0.051. Standardized loadings ranged from 0.57 to 0.84, and all reliability and validity indices are reported in Table 2. Composite reliability ranged from 0.81 to 0.87, and average variance extracted ranged from 0.48 to 0.54; the two values marginally below the 0.50 threshold (engagement and AI literacy) were accompanied by composite reliabilities well above 0.70, supporting convergent validity. The square root of each construct’s average variance extracted exceeded its correlations with the other constructs, supporting discriminant validity. Harman’s single-factor test attributed only 18.1% of variance to a single factor, suggesting that common method variance was not a serious threat, although we note that Harman’s test is a relatively insensitive diagnostic. Figure 3 presents the full structural model with standardized path coefficients.

**Table 2:** Reliability and convergent validity of the measurement model.

Construct	Items	*α*	*CR*	*AVE*	*λ* range
Feedback engagement	5	0.81	0.82	0.48	0.59–0.79
Writing self-efficacy	5	0.84	0.84	0.51	0.66–0.79
Perceived relatedness	4	0.82	0.82	0.54	0.66–0.84
Perceived usefulness	4	0.81	0.81	0.52	0.67–0.75
Intrinsic motivation	6	0.86	0.87	0.52	0.66–0.80
AI literacy	6	0.85	0.86	0.50	0.57–0.76

Model fit: *χ*^2^(390) = 550.85, *χ*^2^/df = 1.41, CFI = 0.952, TLI = 0.946, RMSEA = 0.038, SRMR = 0.051. *α* = Cronbach’s *α*; CR = composite reliability; AVE = average variance extracted; *λ* = standardized factor loading. *N* = 287.

**Figure 3:**
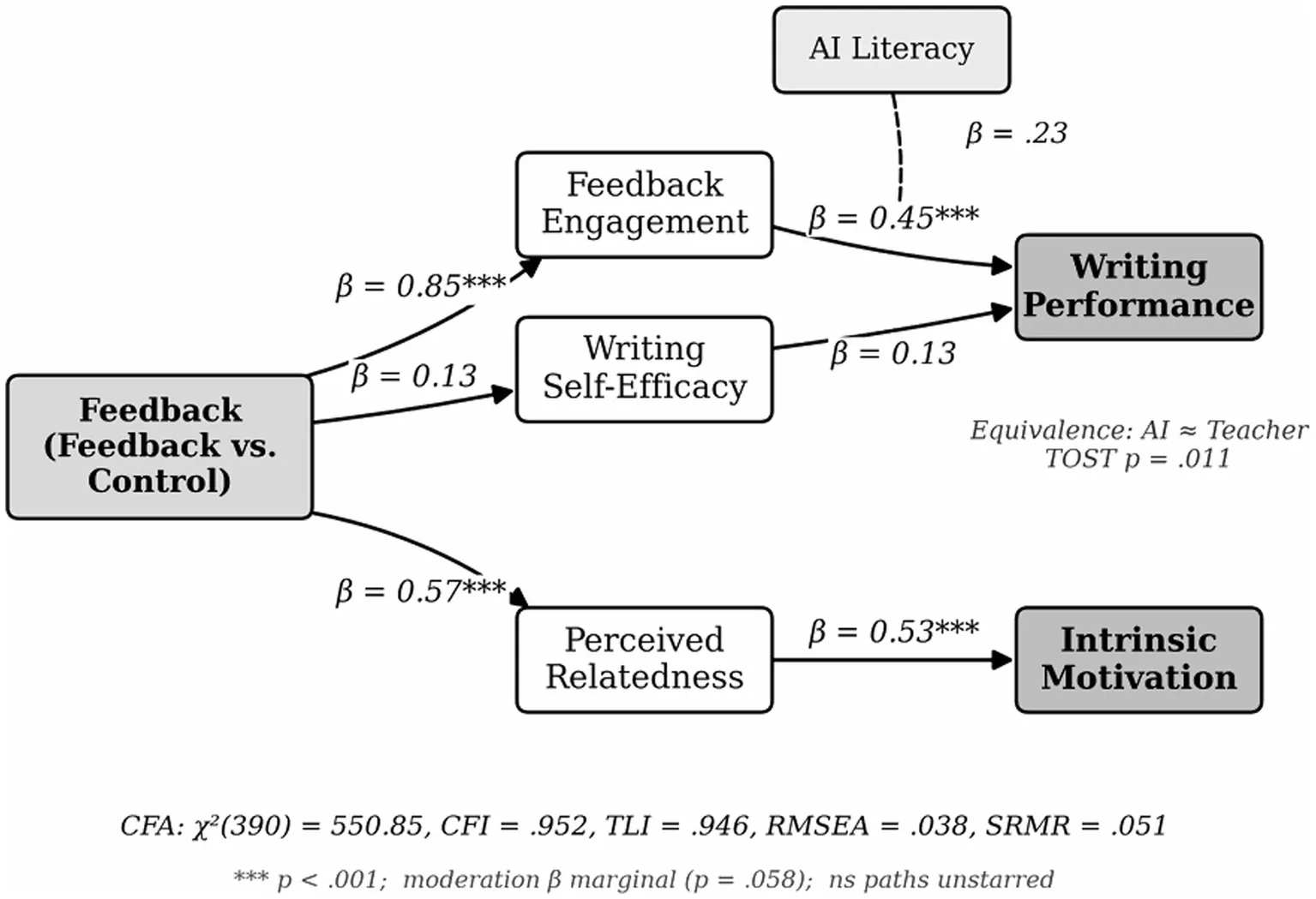
Structural model with standardized path coefficients. Solid paths are significant; unstarred paths are non-significant. The moderation coefficient was not statistically significant (*p* = 0.058) and is shown for exploratory purposes. Fit indices are from the confirmatory factor analysis. ***p < 0.001.

Multi-group CFA supported measurement invariance across the three conditions. The configural model fit adequately by RMSEA and SRMR and marginally by CFI and TLI, as is common when 30 indicators are estimated in groups of fewer than 100 participants, *χ*^2^(1170) = 1449.32, CFI = 0.907, TLI = 0.896, RMSEA = 0.050, SRMR = 0.080. Constraining the factor loadings to equality did not worsen fit, Δ*χ*^2^(48) = 54.15, *p* = 0.252, ΔCFI = −0.002, ΔRMSEA < 0.001, ΔSRMR = 0.005, and neither did additionally constraining the item intercepts, Δ*χ*^2^(48) = 39.35, *p* = 0.809, ΔCFI = +0.003, ΔRMSEA = −0.002, ΔSRMR = 0.001. Scalar invariance was therefore supported, and between-condition differences in the scale scores can be interpreted as differences in the constructs rather than in how the items functioned across conditions.

Students’ ratings suggested that the two feedback streams were broadly comparable from the recipient’s perspective. AI and teacher feedback did not differ in rated usefulness (*M* = 4.82 vs. 4.80), *t*(188) = 0.15, *p* = 0.881, or in the depth of engagement they elicited (*M* = 4.64 vs. 4.66), *t*(188) = −0.19, *p* = 0.849, whereas they differed in perceived relatedness (*M* = 4.12 vs. 4.65), *t*(188) = −4.08, *p* < 0.001. These are recipients’ perceptions rather than an independent coding of the feedback itself (see Section 5.5).

### 4.2 Performance: H1 and H2

Supporting H1, and as summarized in Table 3, the ANCOVA revealed a significant effect of condition on post-test writing, *F*(2, 281) = 11.46, *p* < 0.001, partial *η*^2^ = 0.075, controlling for pre-test writing, GPA, and gender. Adjusted means were 48.28 for control, 54.70 for AI feedback, and 54.58 for teacher feedback. Both feedback conditions outperformed the control by a medium margin: AI versus control, Δ*M* = 6.42, *d* = 0.60, 95% CI [0.32, 0.89], *p* < 0.001; teacher versus control, Δ*M* = 6.29, *d* = 0.59, 95% CI [0.30, 0.88], *p* < 0.001; both contrasts remained significant after Holm adjustment (adjusted *p*s < 0.001). The ANCOVA assumptions were satisfied: the regression slopes of post-test on pre-test writing were homogeneous across conditions (condition × pre-test), *F*(2, 279) = 0.99, *p* = 0.373; residual variances did not differ across conditions, Brown–Forsythe *F*(2, 284) = 1.46, *p* = 0.234; residuals were approximately normal, Shapiro–Wilk *W* = 0.995, *p* = 0.401 (skewness = 0.17, excess kurtosis = −0.20); no standardized residual exceeded |3|; and the largest Cook’s distance was 0.03. With HC3 robust standard errors the conclusions were unchanged (AI vs. control, *t* = 4.11; teacher vs. control, *t* = 4.25; both *p*s < 0.001). Receiving substantive feedback of either kind thus conferred a comparable, educationally meaningful advantage in revised-essay quality relative to the control (Figure 4A). Because the pre-test (a timed first draft) and the post-test (a revised draft) were scored on the same rubric but under different conditions, the pre-test served as a covariate to equate baseline standing, and H1 concerns between-condition differences in revised-essay quality rather than within-person change.

**Table 3:** Effects of feedback condition on writing performance and intrinsic motivation.

Outcome and contrast	Δ*M*	*d*	95% CI	*p*
Writing performance [ANCOVA *F*(2, 281) = 11.46, *p* < 0.001, partial *η*^2^ = 0.075]				
AI vs. control	6.42	0.60	[0.32, 0.89]	<0.001
Teacher vs. control	6.29	0.59	[0.30, 0.88]	<0.001
AI vs. teacher (TOST equivalence)	0.12	0.01	[−2.43, 2.68]^a^	0.011
Intrinsic motivation [ANOVA *F*(2, 284) = 10.96, *p* < 0.001]				
Teacher vs. AI	0.26	0.31	–	0.029
AI vs. control	0.29	0.35	–	0.016
Teacher vs. control	0.55	0.65	–	<0.001

Δ*M* = difference in adjusted means (performance) or raw means (motivation). Adjusted performance means: control = 48.28, AI = 54.70, teacher = 54.58. Motivation means: control = 4.61, AI = 4.90, teacher = 5.16. ^a^The AI vs. teacher comparison used a two one-sided tests (TOST) equivalence procedure with a ± 0.35 SD margin; the 90% CI fell within the bounds (TOST *p* = 0.011). Performance d values are standardized on the ANCOVA residual SD (10.62); motivation d values on the total-sample SD (0.83). Tabled *p* values are unadjusted; all contrasts remained significant after Holm adjustment within each outcome family (adjusted *p* ≤ 0.031).

**Figure 4:**
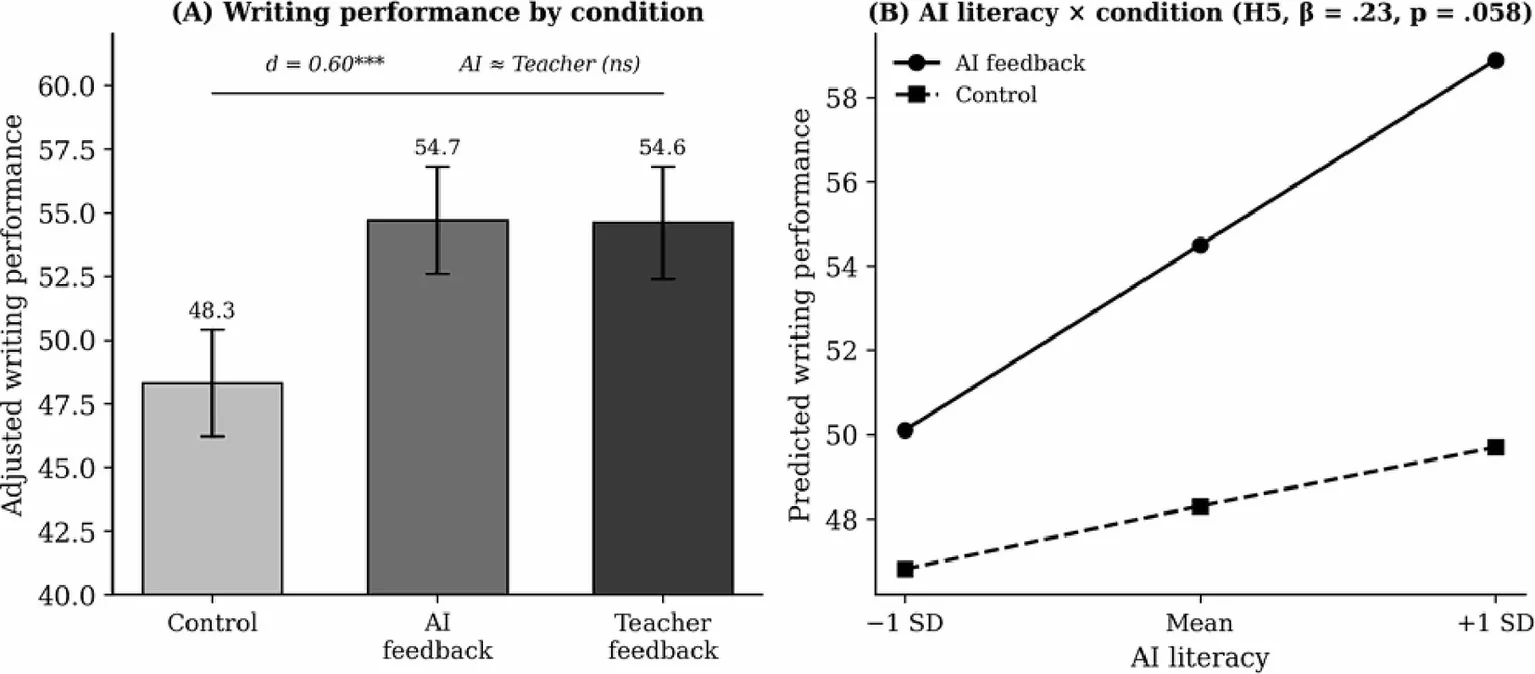
Writing performance by condition and the AI literacy × condition interaction. **(A)** Adjusted writing performance by condition with 95% confidence intervals. **(B)** Simple slopes of AI literacy on predicted writing performance for the AI-feedback and control conditions. Panel **(B)** is exploratory; the focal interaction was not significant (*p* = 0.058).

Revised-essay scores were, on average, lower than first-draft scores in all three conditions (overall *M* = 61.65 vs. 52.49), paired *t*(286) = −12.94, *p* < 0.001. We verified these values against the raw data; they are not a transcription error. The decline was largest in the control condition (mean change = −13.24 points) and about half as large in the AI (−7.09) and teacher (−7.04) conditions. The between-condition effects reported above therefore reflect a smaller decline among students who received feedback, not an absolute improvement over the first draft. Because the raters scored the pre- and post-test essays intermixed and blind to draft status, the decline is unlikely to reflect a shift in scoring standards; it more plausibly reflects the conditions under which revision took place. Revision came at the end of Session 2, after students had read their feedback and completed the questionnaires, and the 40 min allowed for revision were slightly shorter than the 45 min allowed for the first draft, so students who attempted substantial restructuring often could not integrate the changes fully, leaving gaps in coherence and new surface errors. In addition, course credit depended on attendance rather than on essay quality, so the revision carried no external stakes. These conditions applied equally to all three arms; they lowered revised-essay scores overall but do not confound the between-condition comparisons that are the focus of the study. We return to this point in Section 5.5.

Supporting H2, the two feedback conditions were statistically equivalent on performance within the pre-specified margin. The adjusted AI-minus-teacher difference was 0.12 points (*d* = 0.01), and the 90% confidence interval for this difference, [−2.43, 2.68], fell entirely within the equivalence bounds of ±3.72 points (*d* = ±0.35, standardized on the residual *SD* of 10.62); the TOST procedure was significant, *p* = 0.011. This conclusion was robust to a somewhat stricter margin (*d* = 0.30, TOST *p* = 0.024) but not to margins of *d* ≤ 0.25, indicating that AI and teacher feedback are equivalent for differences up to roughly one third of a standard deviation rather than for arbitrarily small ones. The equivalence conclusion therefore applies to revised-essay quality under this margin and this experimental protocol: it indicates that any difference between the two feedback sources on this outcome was smaller than the margin we defined as educationally important, not that the two sources are interchangeable in general.

### 4.3 Mechanisms of the performance effect: H3

A parallel multiple-mediator model tested whether the performance benefit of feedback (feedback conditions vs. control) operated through engagement and self-efficacy; all indirect effects and the moderation results are reported in Table 4. Consistent with H3a, the indirect effect through feedback engagement was significant, indirect = 5.05, 95% CI [3.57, 6.69]: feedback raised engagement (unstandardized *a* = 0.72), and engagement in turn predicted post-test performance (*b* = 7.03 rubric points per scale unit), so that the two paths multiply to the reported indirect effect. Contrary to H3b, the indirect effect through writing self-efficacy was not significant, indirect = 0.23, 95% CI [−0.20, 0.75]; although feedback nominally raised self-efficacy, the path was weak and did not transmit a reliable effect to performance. After accounting for both mediators, the direct effect was small and non-significant, indicating that engagement statistically accounted for much of the between-condition difference in revised-essay quality. H3 was thus partially supported: engagement, but not self-efficacy, carried a significant indirect effect. Because engagement was measured after the manipulation but was not itself manipulated, this indirect effect is consistent with, but does not establish, causal mediation; unmeasured variables that influence both engagement and revision quality could produce the same pattern.

**Table 4:** Indirect effects and moderation results.

Effect	Estimate	95% CI	Decision/*p*
Indirect effects (bootstrap, 5,000 resamples)			
Engagement (performance) (Feedback vs. control)	5.05	[3.57, 6.69]	Significant
Self-efficacy (performance) (Feedback vs. control)	0.23	[−0.20, 0.75]	n.s.
Relatedness (motivation) (Teacher vs. AI)	0.25	[0.12, 0.40]	Significant
Moderation: AI literacy × condition on performance			
AI literacy × AI condition (*β* = 0.23)	3.58	–	*p* = 0.058 (n.s.)
AI literacy × teacher condition (*β* = 0.04)	0.62	–	*p* = 0.442
Interaction block (Δ*R*^2^)	0.015	–	Δ*F*(2, 280) = 3.53, *p* = 0.031

Mediation a- and b-paths (unstandardized): engagement *a* = 0.72, *b* = 7.03; self-efficacy *a* = 0.11, *b* = 2.11; relatedness *a* = 0.53, *b* = 0.48. Simple slopes of AI literacy on performance: control = 1.69, AI feedback = 5.26. *β* = standardized coefficient. *N* = 287. The AI literacy × AI condition interaction was not significant; the simple slopes are reported for exploratory purposes only.

### 4.4 Motivation: H4

Although AI and teacher feedback were equivalent on performance, they diverged on motivation. A one-way ANOVA on intrinsic motivation showed a significant effect of condition, *F*(2, 284) = 10.96, *p* < 0.001, with means of 4.61 (control), 4.90 (AI), and 5.16 (teacher). Teacher feedback produced higher intrinsic motivation than AI feedback, *d* = 0.31, and the largest gap was between teacher feedback and control, *d* = 0.65; AI feedback in turn exceeded the control, *d* = 0.35. Holm-adjusted pairwise comparisons confirmed each difference: teacher versus AI, *t*(188) = 2.20, *p* = 0.029 (adjusted *p* = 0.031); AI versus control, *t*(191) = 2.44, *p* = 0.016 (adjusted *p* = 0.031); teacher versus control, *t*(189) = 4.77, *p* < 0.001 (adjusted *p* < 0.001). Variances were homogeneous across conditions, Brown–Forsythe *F*(2, 284) = 0.03, *p* = 0.968. Supporting H4, a mediation analysis within the two feedback conditions showed that the teacher-versus-AI advantage in motivation was statistically accounted for by perceived relatedness: teacher feedback elicited stronger relatedness (unstandardized *a* = 0.53), which predicted intrinsic motivation (*b* = 0.48), yielding a significant indirect effect, indirect = 0.25, 95% CI [0.12, 0.40], with a negligible direct effect. Because relatedness and intrinsic motivation were measured concurrently, this pathway is best read as associational rather than strictly causal; relatedness was not manipulated, and the indirect effect cannot rule out, for example, that students who enjoyed the task more also reported feeling more connected to the feedback source. To probe the performance-motivation dissociation directly rather than inferring it from two separate models, we modeled the standardized performance and motivation outcomes jointly and estimated the feedback-source (teacher vs. AI) by outcome (performance vs. motivation) interaction; the interaction was in the predicted direction but marginal, *b* = 0.35, *t*(376) = 1.70, *p* = 0.089, consistent with, but not a confirmatory test of, a pattern in which feedback source is largely inconsequential for performance yet matters for motivation; we therefore treat the dissociation as preliminary. Taken together, the motivational premium of teacher feedback was associated with the stronger sense of human connection it conveyed, which AI feedback, although rated as similarly useful, conveyed to a lesser degree.

### 4.5 AI literacy as a hypothesized boundary condition: H5

Finally, we tested whether AI literacy moderated the effect of AI feedback on performance. Adding the AI-literacy-by-condition interactions explained a significant increment of variance, Δ*R*^2^ = 0.015, Δ*F*(2, 280) = 3.53, *p* = 0.031. The focal AI-by-AI-literacy interaction was in the predicted direction but did not reach significance, *b* = 3.58, *β* = 0.23, *p* = 0.058, whereas the teacher-by-AI-literacy interaction was negligible, *p* = 0.442. Exploratory simple-slope analysis (Figure 4B) showed that AI literacy was more strongly associated with performance among students who received AI feedback (slope = 5.26) than among control students (slope = 1.69), suggesting that more AI-literate students may have extracted more benefit from machine feedback, while AI literacy was largely inert for teacher feedback. Because the focal interaction was not significant, H5 was not supported; we report this pattern as exploratory and hypothesis-generating.

## 5 Discussion

This experiment asked whether AI-generated feedback can stand in for a teacher’s feedback on student writing, and it returns a nuanced answer: under the conditions tested, largely yes for revised-essay quality, but not for motivation. Both AI and teacher feedback produced higher revised-essay quality than a no-feedback control by a medium margin, and an equivalence test indicated that AI feedback was as effective as teacher feedback on this performance criterion within a margin of ±0.35 SD. Yet the two sources were not interchangeable. Teacher feedback fostered higher intrinsic motivation than AI feedback, a difference statistically accounted for by students’ sense of relatedness to the feedback source. Mechanistically, the shared performance benefit was associated with depth of engagement rather than with writing self-efficacy, and an exploratory, non-significant pattern suggested that the AI advantage accrued somewhat more to students higher in AI literacy. Below we interpret these findings, draw out their theoretical and practical implications, and acknowledge their limits.

### 5.1 AI feedback matches teacher feedback on revised-essay quality, and why

That AI feedback was equivalent to teacher feedback on revised-essay quality is noteworthy, and the equivalence-testing framing allows this conclusion to be stated more precisely than a non-significant difference test would, while also marking its limits: equivalence was established for differences up to ±0.35 SD, for one argumentative task, in a single feedback-and-revision cycle. Prior work has tended to ask whether AI and human feedback differ in quality, often finding small differences in one direction or another (Banihashem et al., 2024; Steiss et al., 2024). By reframing the comparison as one of equivalence on a learning outcome, we move past the ambiguous non-significant difference and provide positive evidence that, for the revision of an argumentative essay under the conditions of this experiment, machine feedback performed within the margin we defined as educationally negligible. This finding aligns with the processing logic of Feedback Intervention Theory (Kluger and DeNisi, 1996): if what makes feedback effective is the cognitive engagement and task-focused revision it provokes, then the identity of the messenger should be secondary to the quality of the message, provided that quality is matched, which our design approximated by equating length, format, and rubric focus, an approximation consistent with students’ similar usefulness ratings. Our mediation results are consistent with this account. The performance benefit of feedback was statistically accounted for by engagement, the degree to which students processed and acted on comments, and not by self-efficacy. In other words, feedback appears to have benefited revision primarily by giving students something substantive to work with and a reason to work with it, rather than by making them feel more capable in the moment. Where AI feedback is as specific and actionable as a teacher’s, it can engage students as deeply and yield a comparable benefit; whether it does so with other models, prompts, and tasks is an empirical question.

The null mediating role of self-efficacy is itself informative. Self-efficacy theory holds that confidence is built partly through credible persuasion from valued others (Bandura, 1977; Schunk, 1991), a route that a single feedback episode, particularly from a machine, may be poorly positioned to travel. That engagement rather than self-efficacy accounted for the effect suggests that the proximal driver of short-term writing improvement is cognitive-behavioral processing, not a swift change in self-belief. This dovetails with feedback-as-process scholarship that locates the active ingredient in what learners do with feedback rather than in how it makes them feel (Carless and Boud, 2018; Molloy et al., 2019; Nicol, 2020).

### 5.2 A performance-motivation dissociation

The study’s central contribution is the joint pattern it suggests: feedback source made no educationally meaningful difference to revised-essay quality but mattered for motivation. We label this a preliminary performance-motivation dissociation while noting that its most direct test, the source-by-outcome interaction, was only marginal (*b* = 0.35, *p* = 0.089); the dissociation therefore rests chiefly on the conjunction of a significant performance equivalence and a significant motivational difference, with the integrated interaction offering suggestive corroboration that future, better-powered work should confirm. Teacher feedback produced higher intrinsic motivation than AI feedback matched in length, format, and rubric focus, and perceived relatedness statistically accounted for this gap. This is the pattern anticipated when the cognitive channel emphasized by Feedback Intervention Theory and the relational channel emphasized by Self-Determination Theory are considered together. FIT, concerned with the cognitive channel, anticipates equivalence on performance. SDT, concerned with the social-relational channel, anticipates divergence on motivation, because feedback from a person satisfies the need for relatedness in a way that feedback from an algorithm does not (Ryan and Deci, 2000). The same feedback content thus travels two routes with different consequences depending on its source. Our findings give empirical shape to a worry that has so far been mostly anecdotal, that students appreciate the efficiency of AI feedback while still wanting the human connection of a teacher (Barrett and Pack, 2023; Chan and Hu, 2023), and they suggest where the shortfall lies: it is relatedness, not usefulness, on which AI feedback fell short in this study. Notably, AI feedback was not demotivating in absolute terms; it modestly exceeded the control on motivation. The issue is comparative: students found machine feedback about as useful as teacher feedback but drew less relatedness from it, and over the long run such a relational gap may carry motivational costs that a single feedback-and-revision cycle cannot reveal.

This dissociation also speaks to the broader literature on trust in automation, which finds that people extend less benevolence to machine agents than to humans even when the machines perform competently (Araujo et al., 2020). Our results suggest that the educational analogue of that asymmetry is motivational rather than performance-related: students may let a machine help them revise, and benefit from it, but they do not draw the same sense of being cared for from it.

### 5.3 AI literacy: an exploratory pattern and a possible equity concern

The moderation by AI literacy was not statistically significant, and we interpret it as exploratory. The interaction block was significant, but the focal AI-by-AI-literacy term did not reach significance (*p* = 0.058), so H5 was not supported. The pattern was nonetheless in the predicted direction: more AI-literate students appeared to gain more from AI feedback, while AI literacy was inert for teacher feedback. The asymmetry is theoretically coherent, extracting value from machine feedback requires knowing how to interpret, question, and selectively act on it, a competence that AI literacy indexes (Carolus et al., 2023; Ng et al., 2023; Wang et al., 2022), whereas making use of a teacher’s comments is a long-practiced skill that does not depend on AI literacy. Although the evidence here is exploratory rather than conclusive, it raises a possible equity concern that warrants attention: if the benefits of AI feedback flow disproportionately to students who already understand AI, then deploying AI feedback at scale could widen rather than narrow gaps, rewarding the digitally advantaged. This possibility deserves direct, adequately powered study, and it cautions against treating AI feedback as a uniformly distributed good.

### 5.4 Practical implications

For educators weighing AI feedback, our results counsel neither uncritical enthusiasm nor reflexive resistance, but design. If the equivalence observed here on revised-essay quality holds in other settings, AI feedback could be deployed responsibly to address a real constraint, the scarcity of timely, specific feedback, especially in large courses where students might otherwise receive little. But because AI feedback elicited less intrinsic motivation, a difference associated with lower relatedness, it should not simply replace the teacher. The implication is a blended model: let AI supply the high-volume, fast, cognitively substantive feedback that drives engagement and revision, while reserving teacher feedback for the relational, motivational, and higher-order functions that humans uniquely serve. Such a division of labor leverages the comparative advantage of each source, the machine’s scalability and the teacher’s capacity to relate, rather than forcing a choice between them. Just as brief, scalable interventions have produced durable motivational gains under the right conditions (Yeager et al., 2019), a well-designed blend may capture the reach of AI without sacrificing the relational basis of motivation. Two further design moves follow. First, AI feedback might be made more relationally resonant, through framing, personalization, or transparent teacher endorsement, although whether engineered warmth can substitute for genuine relationship is an open question. Second, if the exploratory AI-literacy pattern is confirmed, institutions that adopt AI feedback should pair it with instruction in how to use it, so that its benefits do not accrue only to the already-skilled.

### 5.5 Limitations and future directions

Several limitations qualify these conclusions and chart an agenda for future work. First, the study captured two supervised sessions encompassing a single feedback-and-revision cycle on one argumentative prompt; the performance equivalence and the motivational gap may both evolve over time, and the long-run consequences of a sustained relatedness deficit, on persistence, course engagement, and writing development, cannot be inferred from one episode. Longitudinal and repeated-exposure designs are needed, ideally with multiple sessions built around different argumentative prompts. A crossover design, in which each student receives AI and teacher feedback on different essays in counterbalanced order, would allow the two sources to be compared within persons and would reduce the influence of individual differences, although it would need to address carryover between sessions. Second, motivation and the mediators were self-reported and measured proximally; behavioral and longer-horizon indicators of motivation would strengthen the case. Questionnaires also cannot show how students interpreted the source of their feedback; in-depth interviews or think-aloud protocols, which we did not collect, would help explain why teacher feedback evoked stronger relatedness. The performance outcome, by contrast, was scored by raters blind to condition and draft status. Third, the AI-feedback condition reflects one implementation, a rubric-anchored large language model (Section 3.2), and results may differ for other models, prompts, or interfaces, including systems explicitly designed to be more personable. Relatedly, the two feedback streams were equated on length, format, and rubric criteria, and students rated them as similarly useful, but we did not independently code the feedback for accuracy, specificity, actionability, tone, or rubric alignment; such coding would provide a stronger test of whether the two sources were comparable in content. Fourth, the sample comprised first-year writers at a single institution working on one argumentative task; generalization to other disciplines, genres, proficiency levels, institutions, and educational cultures remains to be established, and broad inferential leaps from any single design warrant caution (Yarkoni, 2020).

Fifth, while the equivalence margin was chosen *a priori* on defensible grounds, smallest-effect-of-interest decisions are inevitably partly conventional, and as our sensitivity analysis showed, equivalence held at margins of *d* = 0.35 and 0.30 but not at *d* ≤ 0.25, so readers applying a stricter margin should interpret the equivalence claim accordingly. The TOST procedure also had modest power (0.55 at the ±0.35 SD margin), so the equivalence finding should be replicated in a larger sample. Sixth, revised drafts scored lower than first drafts in all three conditions (Section 4.2); the feedback effects are therefore relative to a control group whose scores declined more, and they should not be read as evidence that feedback raised essays above their first-draft level. Seventh, the analyses were based on complete cases; attrition was low and similar across arms, but an intention-to-treat analysis was not conducted. A further caveat concerns the social framing of the manipulation: because students knew whether their feedback came from a person or a machine, the relatedness difference may partly reflect expectations attached to the source rather than an intrinsic property of machine feedback, and a source-blind design would help adjudicate this. The no-feedback control, moreover, did not separate feedback from re-exposure to the rubric criteria, so the performance advantage of feedback is best read as the benefit of essay-specific commentary over minimal prompting. Although the study was powered for the primary performance contrast, the equivalence, within-feedback-group mediation, and moderation tests were comparatively under-powered, one reason we treat the AI-literacy result as provisional. Finally, the moderation by AI literacy was not statistically significant and requires confirmation in studies powered specifically to detect it. In addition, because the mediators were not randomized and relatedness and motivation were measured on the same occasion, the indirect effects are associational; designs that manipulate relatedness directly, for example by varying whether a teacher endorses AI-generated comments, would provide a stronger causal test. Future research should test blended feedback designs head to head, examine whether AI feedback can be engineered to support relatedness without misleading students about its source, and follow the equity question of who benefits from AI feedback over time.

## 6 Conclusion

As generative AI moves into the writing classroom, the temptation is to ask simply whether it is as good as a teacher, as if learning had a single criterion. This experiment shows why that question is too blunt. Judged by revised-essay quality, and within the equivalence margin and controlled protocol of this study, AI feedback performed comparably to teacher feedback: students who received machine feedback revised their essays about as well as those who received human feedback, and better than those who received none, with the benefit associated with the depth of engagement that good feedback provokes. Judged by motivation, the two sources parted ways: teacher feedback nourished intrinsic motivation more than AI feedback did, a difference associated with the stronger sense of relatedness it conveyed. AI feedback was rated about as useful as a teacher’s, but students drew less of a sense of relatedness from it, and that distinction may matter for how learning is sustained. These conclusions come from a single feedback-and-revision cycle with first-year students at one institution writing one argumentative essay, and they await replication across sessions, tasks, and settings. The constructive response is not to choose between human and machine but to combine them deliberately, assigning to AI the scalable, cognitive work it does well and to teachers the relational work that keeps students wanting to write. Used this way, AI feedback may be best understood not as a replacement for the teacher but as an amplifier of what teachers have never had enough of: time to give every student feedback that helps them improve.

## Data Availability

The original contributions presented in the study are included in the article/supplementary material, further inquiries can be directed to the corresponding author.
