# Supplementary material for: As effective as the teacher, but less motivating: a randomized controlled comparison of AI-generated and teacher feedback on students’ writing performance and intrinsic motivation
Source: Front Psychol. 2026 Oct 30;17:1918748. doi: 10.3389/fpsyg.2026.1918748 (PMC13759775; doi:10.3389/fpsyg.2026.1918748)
Supplement: Supplementary file 1 [file Supplementary_file_1.docx]

**Supplementary Material**

*As effective as the teacher, but less motivating: a randomized controlled comparison of AI-generated and teacher feedback on students’ writing performance and intrinsic motivation*

Ying Liu and Mo Shi

**Supplementary Appendix A. Analytic Rubric Used to Score the Pre- and Post-Test Essays**

The analytic rubric evaluates argumentative essays on four equally weighted criteria, each scored from 0 to 25, for a total of 100 points.

| **Criterion** | **Score band** | **Descriptor** |
| --- | --- | --- |
| Thesis and Argument (0–25 points) | 21–25 | Thesis is clearly stated, specific, and debatable; argument is fully developed with compelling reasoning. |
|  | 16–20 | Thesis is clear and arguable; argument is developed but may lack depth in places. |
|  | 11–15 | Thesis is present but vague or overly broad; argument is partially developed. |
|  | 6–10 | Thesis is unclear or descriptive rather than argumentative; reasoning is weak. |
|  | 0–5 | No identifiable thesis; no coherent argument. |
| Organization and Structure (0–25 points) | 21–25 | Essay is logically organized with effective transitions; introduction, body, and conclusion are well developed. |
|  | 16–20 | Organization is generally clear; transitions are present but occasionally mechanical. |
|  | 11–15 | Organization is recognizable but uneven; some paragraphs lack clear purpose. |
|  | 6–10 | Organization is difficult to follow; paragraphs are poorly connected. |
|  | 0–5 | No discernible organizational plan. |
| Evidence and Support (0–25 points) | 21–25 | Claims are supported with specific, relevant evidence; counterarguments are acknowledged and addressed. |
|  | 16–20 | Evidence is relevant and mostly specific; counterarguments are acknowledged but underdeveloped. |
|  | 11–15 | Some evidence is provided but may be general or loosely connected to claims; counterarguments are not addressed. |
|  | 6–10 | Evidence is sparse, irrelevant, or anecdotal; no engagement with opposing views. |
|  | 0–5 | No evidence offered. |
| Language and Mechanics (0–25 points) | 21–25 | Writing is fluent, precise, and virtually error-free; academic register is appropriate throughout. |
|  | 16–20 | Writing is clear with minor errors that do not impede understanding; register is generally appropriate. |
|  | 11–15 | Writing is understandable but contains noticeable grammatical or lexical errors; register is inconsistent. |
|  | 6–10 | Frequent errors impede comprehension; word choice is often inappropriate. |
|  | 0–5 | Writing is largely incomprehensible. |

**Supplementary Appendix B. Prompt Used to Generate the AI Feedback**

The following prompt was submitted to Qwen2.5-27B-Instruct (temperature = 0.7; max_tokens = 1,024). Each student’s de-identified essay was appended after the prompt text below.

You are an experienced academic writing instructor. A first-year university student has written the following argumentative essay for a writing course. Please provide detailed, constructive feedback on the essay, organized by the following four criteria: (1) Thesis and Argument: Is the thesis clearly stated, specific, and debatable? Is the argument well developed with sound reasoning? (2) Organization and Structure: Is the essay logically organized? Are transitions effective? Does it have a clear introduction, body, and conclusion? (3) Evidence and Support: Are claims supported with specific, relevant evidence? Are counterarguments acknowledged and addressed? (4) Language and Mechanics: Is the writing fluent and precise? Are there grammatical or lexical errors? Is the academic register appropriate? For each criterion, identify specific strengths and areas for improvement, quoting or referring to specific passages in the essay. Provide concrete suggestions for how the student can revise and improve the essay. Be encouraging but honest. Do not rewrite the essay; focus on guiding the student’s revision. Keep your feedback to approximately 300 words.
